# Identification of gene expression signatures associated with neuroinflammation in discogenic sciatica using machine learning and experimental validation

**DOI:** 10.3389/fgene.2026.1666639

**Published:** 2026-03-05

**Authors:** Fei Jiang, Yang Xu, Xi-Hong Ye, Bin Zheng, Guang-Lei Zhang, Ren-Hu Li

**Affiliations:** 1 Department of Anesthesiology, Lu’an Hospital of Anhui Medical University, Lu’an, Anhui, China; 2 Department of Anesthesiology, Xiangyang Central Hospital, Affiliated Hospital of Hubei University of Arts and Science, Xiangyang, Hubei, China; 3 Department of Pain, Xiangyang Central Hospital, Affiliated Hospital of Hubei University of Arts and Science, Xiangyang, Hubei, China

**Keywords:** Bioinformatics, differentially expressed genes, machine learning, Neuroinflammatory, discogenic sciatica

## Abstract

**Background:**

Sciatica is a debilitating condition characterized by pain radiating along the sciatic nerve, often manifesting due to underlying neuroinflammatory processes. Understanding the molecular mechanisms linking neuroinflammation to sciatica is essential for developing targeted therapeutic interventions. Recent studies have suggested that specific neuroinflammatory genes may play a pivotal role in the pathophysiology of sciatica, offering a potential avenue for understanding this condition.

**Methods:**

This study aimed to elucidate the contributions of neuroinflammatory genes to the development of sciatica. We used publicly available datasets GSE124272 and GSE150408 from the Gene Expression Omnibus (GEO) database of the National Center for Biotechnology Information. By thoroughly analyzing the expression matrices, we identified differentially expressed genes (DEGs) linked to neuroinflammatory pathways. Functional annotation was performed using Gene Ontology (GO) analysis and Gene Set Enrichment Analysis (GSEA). To enhance predictive modeling, we employed Least Absolute Shrinkage and Selection Operator (LASSO) regression and Support Vector Machine Recursive Feature Elimination (SVM-RFE) methods to assess neuroinflammatory gene expression. Lastly, we employed quantita-tive real-time PCR (qRT-PCR) to validate our results.

**Results:**

The analysis revealed that the identified DEGs are significantly enriched in multiple biological pathways relevant to neuroinflammatory responses in patients with sciatica. Notably, LASSO regression and SVM techniques identified four key neuroinflammatory genes: *KLRK1*, *LRRK2*, *NLRP3*, and *PLG*. A bar graph was generated to illustrate the predictive weights of these genes concerning sciatica risk, further complemented by immune cell composition analysis via CIBERSORTx, which underscored significant correlations between these genes and the abundance of various immune cell types in affected individuals.

**Conclusion:**

Our findings substantiate the critical roles of *KLRK1*, *LRRK2*, *NLRP3*, and *PLG* in the neuroinflammation-associated pathogenesis of sciatica, providing pivotal insights into the biological underpinnings of this condition. These neuroinflammatory genes serve as promising targets for advancing therapeutic strategies for sciatica management.

## 1 Introduction

Sciatica, characterized by radiating leg pain, is a significant cause of global disability, with a lifetime prevalence up to 43% and most cases stemming from lumbar disc herniation (Khallaf, 2017). The pathophysiology extends beyond mechanical nerve root compression to encompass a robust neuroinflammatory response. This response, involving immune cell infiltration (e.g., macrophages), glial cell activation, and the release of mediators like IL-1β and TNF-α, is now established as a central driver of pain sensitization and chronicity (Claudia et al., 2018; Hu et al., 2023). Despite advances in understanding these inflammatory components, a significant proportion of patients experience refractory pain, underscoring the limitations of current diagnostic and therapeutic paradigms and highlighting the need for a more integrated molecular understanding (Ma et al., 2021).

To date, research has predominantly focused on elucidating the role of specific, high-profile inflammatory genes and pathways, such as the NLRP3 inflammasome or IL-6 signaling (Zhang et al., 2023; Bhatia et al., 2017). While these studies provide crucial mechanistic insights, they inherently offer a fragmented view. A critical and persistent knowledge gap is the lack of a systematic, genome-wide perspective to define the coordinated gene expression programs and the interactive molecular networks that collectively underpin sciatica pathogenesis. This gap directly limits the development of robust, multi-gene biomarker signatures capable of capturing the complex neuroinflammatory state, which remains an unmet clinical need.

To bridge this gap, we employed an integrated analytical strategy combining transcriptomic data, bioinformatics, and machine learning. This approach is uniquely positioned to address the limitations of single-gene studies by enabling an unbiased, system-level analysis of the transcriptome. Specifically, machine learning algorithms can identify subtle, coordinated patterns across hundreds of genes that might be missed in conventional analyses. Our study aims to leverage this power to identify novel, multi-gene expression signatures associated with neuroinflammation in discogenic sciatica, thereby providing a more holistic framework for understanding disease mechanisms and discovering potential diagnostic biomarkers.

## 2 Materials and methods

### 2.1 Data source

The gene expression datasets GSE124272 and GSE150408 were obtained from the Gene Expression Omnibus (GEO) hosted by the National Center for Biotechnology Information (https://www.ncbi.nlm.nih.gov/geo/) as detailed in Table 1. The original GEO records defined the patient selection criteria for datasets GSE150408 and GSE124272 as follows. The sciatica groups comprised individuals diagnosed with either sciatica or lumbar disc prolapse, both confirmed by magnetic resonance imaging (MRI). The healthy control groups consisted of volunteers without low back pain or sciatica. To maximize data utility for model development while ensuring an unbiased performance estimate, we employed a pre-defined, dataset-level analytical strategy: data from both GSE124272 and GSE150408 were integrated for model development and feature selection, while the entire GSE150408 cohort was reserved as a fixed, hold-out internal validation set for final model evaluation. The gene expression profiling in this study was based on data generated using the Agilent-072363 SurePrint G3 Human GE v3 8 × 60K Microarray platform (GPL21185). The GSE124272 dataset was extracted and divided into 8 healthy normal individuals and 8 individuals with sciatica. For the GSE150408 dataset, we specifically used the baseline (pre-treatment) samples, comprising 17 cases from the normal group and 17 from the sciatica group, prior to any therapeutic intervention. The GSE150408 dataset provided 17 cases from the normal group and 17 from the sciatica group. The pre-processed “Series Matrix” files (e.g., GSE124272_series_matrix.txt) were downloaded from GEO. These files contain background-corrected and summarized expression values. After integrating the datasets from GSE124272 and GSE150408, probes were annotated using the platform-specific annotation file (GPL21185). Quantile normalization was then performed using the normalizeBetweenArrays function from the limma package (version 3.58.1) to ensure comparability across all samples (Ritchie et al., 2015). Subsequently, to adjust for technical batch effects between the two studies, the ComBat function from the sva package (version 3.46.0) was applied to the normalized data using the parametric empirical Bayesian framework (par.prior = TRUE) (Leek et al., 2012). Principal-component analysis was performed on the merged expression matrix before and after ComBat; samples were visualised by dataset to inspect clustering patterns. The proportion of variance attributable to batch was quantified as the ratio of the between-dataset sum of squares to the total sum of squares on the first two PCs (PC1 + PC2). The resulting batch-corrected expression matrix was used for all downstream analyses. Genes associated with neuroinflammatory mechanisms were identified by querying the term “neuroinflammation” across all annotation sections (“All Sections”) and restricting to the “Protein Coding” category in the GeneCards database (https://www.genecar-ds.org/, accessed on May 17, 2025). An initial broad set of genes was obtained from this query, to which a stringent relevance score filter (>1.5) was applied, yielding the final gene set used for subsequent analysis.

**TABLE 1:** Overview of gene expression datasets from sciatica patients' peripheral blood.

GEO accession	GPL platform	Sequencing type	Species	Tissue source	Group information	Clinical context	Sample processing	Primary reference
GSE 124272	GPL 21185	Transcriptomics	Human	Peripheral blood	8 normal cases 8 sciatica cases	Lumbar disc prolapse patients vs. healthy volunteers	PAXgene blood RNA tubes, agilent 8 × 60K array	Wang et al. Exp Ther Med 2019
GSE 150408	GPL 21185	Transcriptomics	Human	Peripheral blood	17 normal cases 17 sciatica cases	Sciatica patients receiving standardized TCM regimen	PAXgene blood RNA tubes, agilent 8 × 60K array	Wang et al. BMC Neurol 2021

### 2.2 Screening of differentially expressed sciatica genes and neuroinflammatory related gene modules

We screened for differentially expressed genes (DEGs) that were significantly expressed in both the control and sciatica groups. Genes with logFC >0.58 and *p* < 0.05 are considered upregulated genes. Conversely, genes with logFC <−0.58 and *p* < 0.05 are classified as downregulated. The chosen criteria for gene expression analysis are |log2(FC)| >0.58 and *p* < 0.05, as shown in a volcano plot. Subsequently, we employed the pheatmap package (version 1.0.13) to illustrate and evaluate gene expression differences between the normal and the sciatica groups.

### 2.3 GO functional enrichment and GSEA enrichment analysis

The analysis of Gene Ontology (GO) functional annotations for DEGs related to neuroinflammation was performed using the “clusterProfiler” package (version 4.0) in the R programming environment (Yu et al., 2012). The gene set ‘c2. cp.v7.5.1. symbol-s. gmt’ was sourced from the Molecular Signatures Database (MSigDB, version 2025.1. Hs, accessed on May 17, 2025) and subsequently employed to conduct Gene Set Enrichment Analysis (GSEA) on the identified DEGs (Liberzon et al., 2015).

### 2.4 Construction of disease risk models

Machine learning analysis followed a pre-defined, two-stage workflow (development/tuning and final evaluation) to ensure robustness. Prior to model training, all feature variables were centered and scaled to a zero mean and unit variance. In the first stage (model development and feature selection), expression data from the integrated GSE124272 and GSE150408 cohorts were pooled. Model development and tuning were conducted using k-fold cross-validation on this combined dataset. For least absolute shrinkage and selection operator (LASSO) regression, performed using the “glmnet” package (version 4.1) (Friedman et al., 2010), the optimal regularization parameter (λ) was determined through 10-fold cross-validation with a fixed random seed (seed = 2022). For feature selection, we employed the Support Vector Machine-Recursive Feature Elimination (SVM-RFE) methodology (Duan et al., 2005) with a radial basis function kernel; its hyperparameters (cost = 0.5, gamma = 0.015625) were optimized via 5-fold cross-validation with a fixed random seed (seed = 2024). The overlapping key genes identified by both methods were extracted using the “Venn” package. In the second stage (final model evaluation), the diagnostic model was constructed using only the consensus hub genes identified above. This final model was then applied, for the first and only time, to the pre-specified, hold-out GSE150408 validation cohort to generate unbiased performance metrics. Calibration curve analysis was performed using the rms package (version 6.4-0) and ResourceSelection package (version 0.3-5). Decision curve analysis was performed utilizing the rmda package (version 1.6) (Vickers and Elkin, 2006). The area under the receiver operating characteristic curve (AUC) and its 95% confidence interval (CI) for the model (Figure 7) were calculated using the pROC R package (version 1.18.0), which implements DeLong’s method. All statistical analyses were performed using R version 4.2.1.

### 2.5 Immune cell infiltration analysis of sciatica

CIBERSORTx employs linear support vector regression to decipher transcriptomic expression matrices, enabling the estimation of the composition and relative abundance of immune cell types within a sample mixture. The gene expression matrix data were analyzed using the standard CIBERSORT algorithm with the LM22 signature matrix (547 genes defining 22 immune cell types) and the default setting of 1 permutation for significance analysis (Newman et al., 2019). This method estimates immune cell fractions, retaining only samples with a CIBERSORT inference p-value <0.05 to ensure reliable deconvolution results, thereby generating a final immune cell infiltration matrix. The R software package “ggplot2” (version 3.4.2) was used to produce hist-ograms illustrating the distribution of immune cell types within each sample. To evaluate the association between immune cell infiltration and DEGs, the Spearman correlation coefficient used to generate a correlation heatmap. Additionally, to explore disparities in immune cell infiltration across groups, boxplots were constructed, aiding the identification of immune infiltration patterns associated with sciatica. To ensure robustness, we performed cross-validation using the MCP-counter algorithm.

### 2.6 mRNA-miRNA, mRNA-TF, and mRNA-drug network construction

We retrieved miRNAs linked to key genes from the TargetScanHuman database (https://www.targetscan.org/vert_80/) and subsequently constructed a regulatory network depicting interactions between mRNAs and miRNAs. We used the ChIPBase v3.0 database (https://rnasysu.com/chipbase3/index.php) to identify transcription factors that bind to hub genes. We constructed an interaction network linking key genes to transcription factors (TFs) after retrieving relationships between TF targets and DEGs. To identify potentially repurposable drugs, we queried DGIdb (version 5.0) using our four hub genes (*PLG*, *LRRK2*, *NLRP3*, *KLRK1*). We applied the “Approved” drug status filter available in the database interface to prioritize clinically translatable agents. Cytoscape software (version 3.9.1) was then used to visualize the mRNA-miRNA regulatory network along with the mRNA-TF and mRNA-drug interaction networks.

### 2.7 Quantitative real-time PCR (qRT-PCR)

Peripheral blood samples were obtained from 15 patients with MRI-confirmed lumbar disc herniation presenting radiating leg pain persisting >3 months and scheduled for lumbar discectomy (8 male/7 female, age 60.2 ± 6.8 years), along with 15 age- and sex-matched healthy controls (age 63.9 ± 7.2 years) without history of chronic pain or neurological disorders. Total RNA was extracted from ≤200 μL whole blood using the EasyPure® miRNA Kit (TransGen Biotech). RNA concentration and purity (A260/280) were measured with a NanoVue Plus spectrophotometer (Cytiva). Reverse transcription was performed with 1 μg total RNA using the Evo M-MLV RT Mix Kit with gDNA Clean for qPCR Ver.2 (Accurate Biology) in a 20 μL reaction (37 °C, 15 min; 85 °C, 5 s). qPCR was run on a QuantStudio 1 system (Thermo Fisher) with PerfectStart® Green qPCR SuperMix (TransGen Biotech). Each 20 μL reaction contained 10 μL SuperMix, 2 μL cDNA (40× diluted), 0.4 μM each primer, and nuclease-free water. Cycling conditions: 94 °C for 2 min; 45 cycles of 94 °C for 5 s and 60 °C for 30 s; melt-curve from 65 °C to 95 °C (0.5 °C/5 s). Gene expression was normalized to ACTIN and calculated using the ΔΔCt method. All primers (designed with Primer-BLAST, amplicons 80–150 bp, Tm ≈ 60 °C) are listed in Supplementary Table S1.

### 2.8 Statistical analysis

**FIGURE 1:**
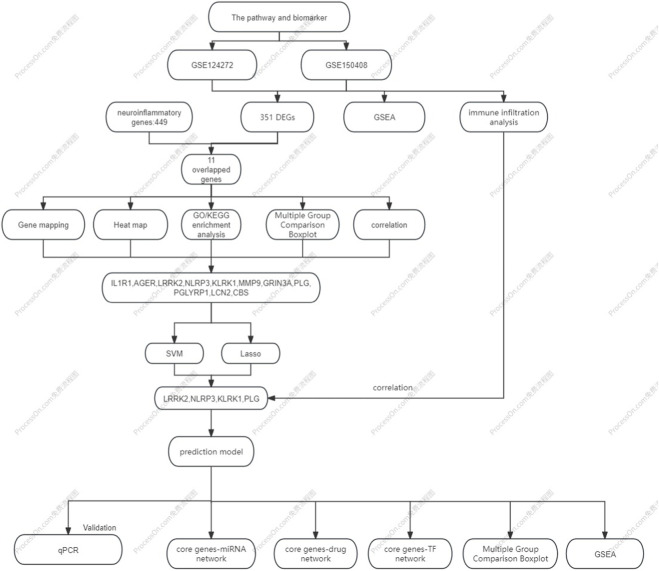
Flow chart of study design and methodology.

Data calculations and analyses were performed using R (version 4.3.3). The normality of continuous variables was assessed using the Shapiro-Wilk test, and the homogeneity of variances was evaluated using Levene’s test. Based on these results, comparisons between two groups for continuous variables were conducted with the Student’s t-test or the Mann-Whitney U test, with p-values <0.05 considered statistically significant. To control for multiple hypothesis testing in high-throughput analyses, the Benjamini–Hochberg false discovery rate (FDR) correction was applied to the p-values from the genome-wide differential expression analysis. In contrast, for the Gene Ontology (GO) functional enrichment analysis and Gene Set Enrichment Analysis (GSEA), the algorithm-generated FDR-corrected q-values are reported, with the complete FDR-adjusted results provided in the supplementary materials for full transparency (Supplementary Table S2). Data from qRT-PCR are presented as mean ± standard deviation (SD). Differences in gene expression levels between the sciatica patient group and the healthy control group were assessed for statistical significance using the two-tailed Student’s t-test. *P* < 0.05 was considered statistically significant. The overall study design is summarized in Figure 1.

## 3 Results

### 3.1 Screening of differentially expressed genes

**FIGURE 2:**
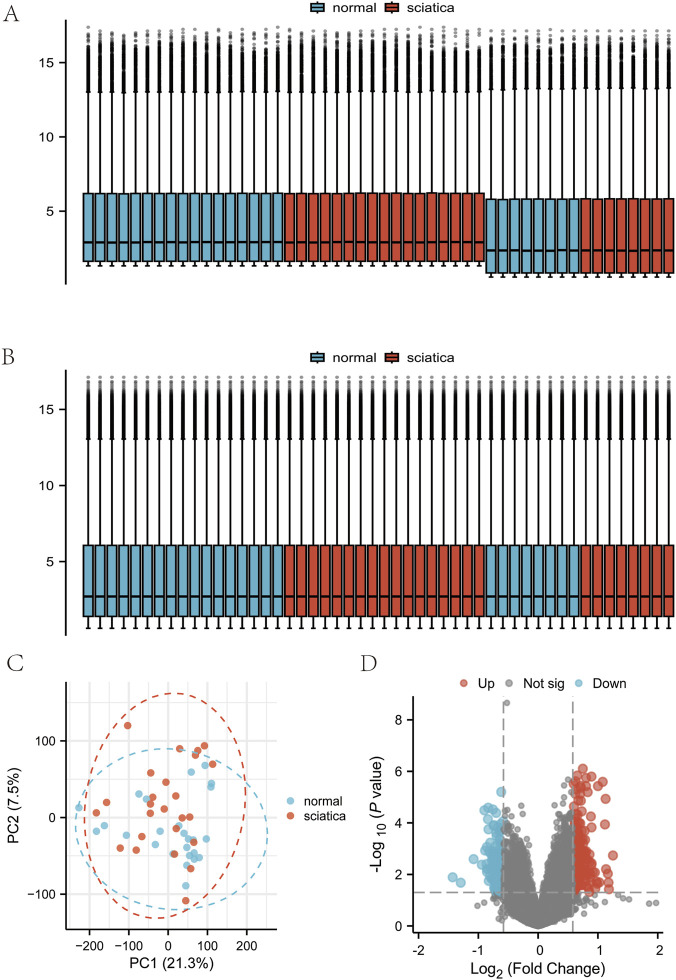
Analysis of differentially expressed genes in the dataset. **(A)** Distribution of expression profiles between samples from the twodata sets before correction. **(B)** the distribution of expression profiles between the two data sets after correction. **(C)** Principal component analysis diagram between the sciatica group and the normal group following batch effect removal. **(D)** Volcano plot based on differential gene analysis.

**FIGURE 3:**
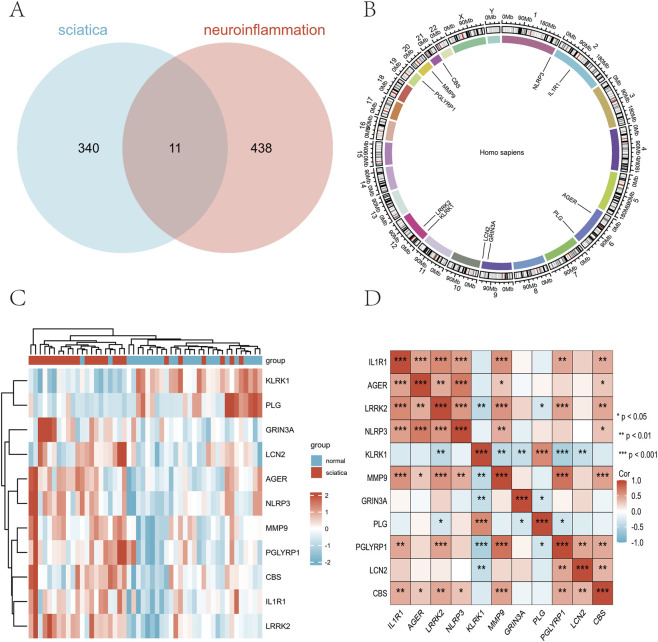
Differentially expressed genes related to neuroinflammatory between healthy controls and sciatica patients. **(A)** Venn diagram identifies 11 neuroinflammatory-associated DEGs in sciatica. **(B)** Chromosomal location distribution of differentially expressed neuroinfla-mmatory related genes. **(C)** Heat map of neuroinflammatory-associated DEGs in sciatica. **(D)** Correlation analysis between differentially expressed neuroinflammatory genes.

After processing the dataset, we obtained 25 samples from the healthy normal group and 25 samples from the sciatica group. The distributions of the data were plotted before and after normalization (Figures 2A,B). Before batch adjustment, the first two principal components (PC1 = 28.1%, PC2 = 9.8%) displayed pronounced dataset-specif- ic segregation that explained 16.2% of the total variance (Supplementary Figure S1; Supplementary Table S3), indicating substantial technical bias. After ComBat correction, this batch-associated variance fell to 2.1% and the dataset clusters vanished (Figure 2C; Supplementary Table S4). We identified 449 neuroinflammation-related genes from GeneCards (https://www.genecards.org/). The combined MSigDB neuroinflammation keyword search resulted in a reference set of 185 unique genes. Notably, 94% of these genes (174 out of 185) were also present in our GeneCards-derived set (Supplementary Table S5). This striking overlap strongly supports the comprehensiveness and specific biological relevance of our initial gene selection for studying neuroinflammation. Principal Component Analysis of the samples from both groups revealed distinct variations in the distribution patterns of 351 associated genes. The differentially expressed genes were shown using volcano plots, with 206 genes upregulated and 145 genes downregulated (Figure 2D). We identified 11 DEGs related to neuroinflammation that differed significantly between sciatica patients and healthy controls (Figure 3A). The chromosomal locations of the eleven DEGs are shown in Figure 3B. In contrast, Figure 3C presents a heatmap of neuroinflammation gene expression differences between the sciatica and normal groups, and Figure 3D shows the correlation among these genes using Spearman correlation analysis.

### 3.2 GO functional enrichment and GSEA enrichment analysis of DEGs

**FIGURE 4:**
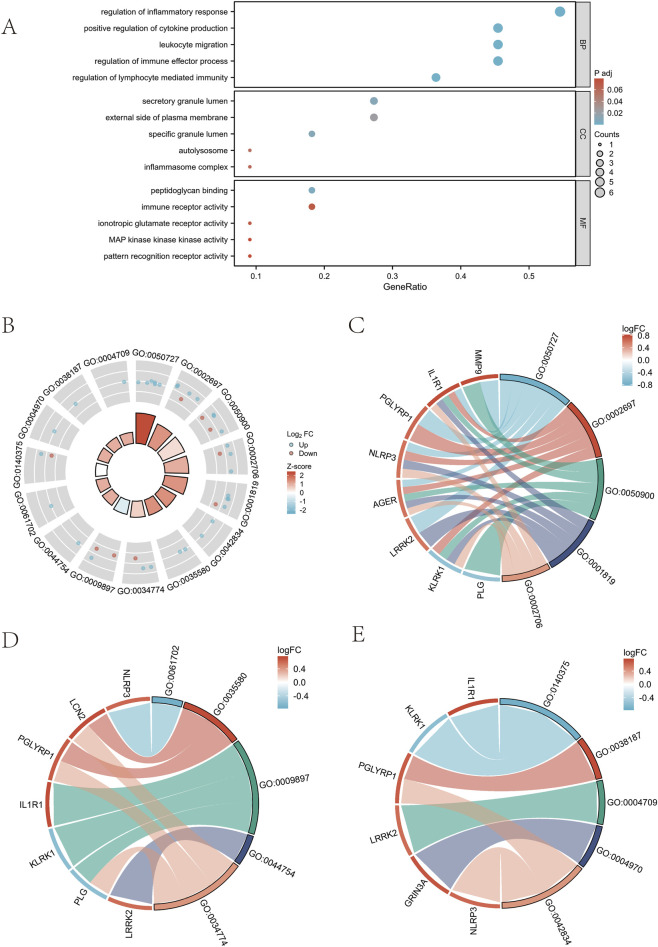
GO enrichment analysis. **(A)** Dot plot of GO enrichment analysis of sciatica-related neuroinflammatory genes. **(B)** The visualization results of GO enrichment analysis circle diagram of sciatica-related neuroinflammatory genes. **(C–E)** are BP(Biological Process), CC(Cellular Component), MF(Molecular Function) functional enrichment chordplots respectively.

**FIGURE 5:**
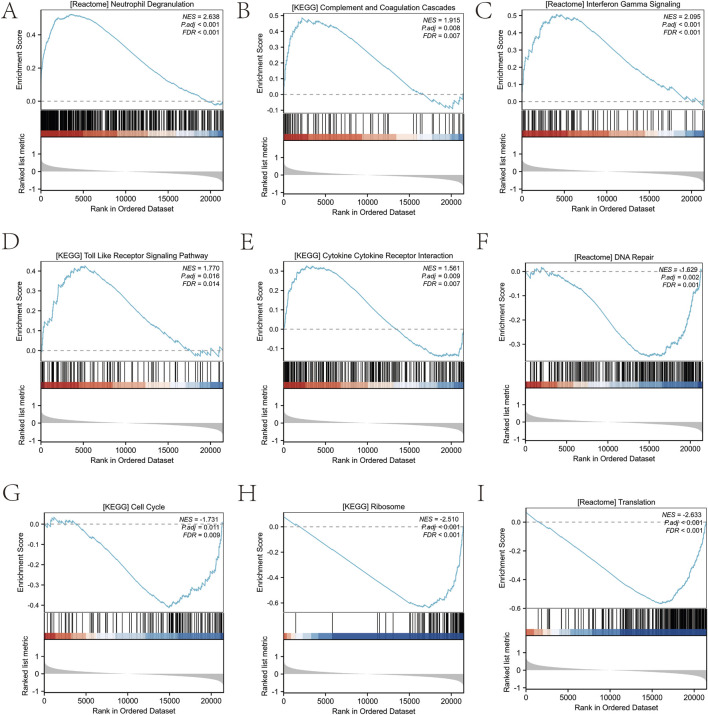
GSEA method was used to rank the effects of different biological pathways. **(A)** GSEA results of REACTOME neutrophil degranulation. **(B)** GSEA results of KEGG complement and coagulation cascades. **(C)** GSEA results of REACTOME interferon gamma signaling. **(D)** GSEA results for KEGG toll like receptor signaling pathway. **(E)** GSEA results for KEGG cytokine-cytokine receptor interaction. **(F)** GSEA results for REACTOME DNA repair. **(G)** GSEA results for KEGG cell cycle. **(H)** GSEA results for KEGG ribosome. **(I)** GSEA results for REACTOME translation.

GO analysis of differentially expressed neuroinflammation genes in sciatica is shown in Table 2. The GO analysis indicated enrichment in various biological processes, including: inflammatory response, immune effector processes, leukocyte migration, cytokine production, and lymphocyte-mediated immunity (Figures 4A–C). Additionally, it highlighted the following cellular components: the inflammasome complex, the specific granule lumen, the external side of the plasma membrane, the autolysosome, and the secretory granule lumen (Figures 4A,B,D). It also identified various molecular functions: immune receptor activity, pattern recognition receptor activity, MAP kinase kinase kinase activity, ionotropic glutamate receptor activity, and peptidoglycan binding (Figures 4A,B,E). Table 3 presents the GSEA results related to sciatica. GSEA results showed positive enrichment of genes in the REACTOME neutrophil degranulation pathway (Normalized Enrichment Score (NES) = 2.638, FDR <0.001) (Figure 5A) and the Kyoto Encyclopedia of Genes and Genomes (KEGG) complement and coagulation cascades pathway (NES = 1.915, FDR <0.001) (Figure 5B). REACTOME interferon gamma signaling (NES = 2.095, FDR <0.001) (Figure 5C), KEGG toll-like receptor signaling pathway (NES = 1.770, FDR <0.001) (Figure 5D), and KEGG cytokine-cytokine receptor interaction (NES = 1.561, FDR <0.001) (Figure 5E). In contrast, negative enrichment was observed in several pathways, including REACTOME DNA repair (NES = −1.629, FDR <0.001) (Figure 5F), KEGG cell cycle (NES = −1.731, FDR <0.001) (Figure 5G), and KEGG ribosome (NES = −2.510, FDR <0.001) (Figure 5H), REACTOME translation (NES = −2.633, FDR <0.001) (Figure 5I). The findings indicate that the expression levels of various neuroinflammation genes are significantly associated with both the initiation and advancement of sciatica.

**TABLE 2:** Gene ontology analysis of differentially expressed glycolysis genes in sciatica.

Ontology	ID	Description	Gene ratio	Bg ratio	p value	p. Adjust
BP	GO:0050727	Regulation of inflammatory response	6/11	394/18800	3.44E-08	2.98E-05
BP	GO:0002697	Regulation of immune effector process	5/11	353/18800	9.55E-07	0.0004
BP	GO:0050900	Leukocyte migration	5/11	384/18800	1.44E-06	0.0004
BP	GO:0001819	Positive regulation of cytokine production	5/11	475/18800	4.10E-06	0.0005
BP	GO:0002706	Regulation of lymphocyte mediated immunity	4/11	175/18800	2.27E-06	0.0004
CC	GO:0061702	Inflammasome complex	1/11	18/19594	0.0101	0.0617
CC	GO:0035580	Specific granule lumen	2/11	62/19594	0.0005	0.01055
CC	GO:0009897	External side of plasma membrane	3/11	455/19594	0.0017	0.0234
CC	GO:0044754	Autolysosome	1/11	11/19594	0.0061	0.0557
CC	GO:0034774	Secretory granule lumen	3/11	322/19594	0.0006	0.0105
MF	GO:0140375	Immune receptor activity	2/11	148/18410	0.0033	0.0707
MF	GO:0038187	Pattern recognition receptor activity	1/11	26/18410	0.0154	0.0790
MF	GO:0004709	MAP kinase kinase kinase activity	1/11	26/18410	0.0154	0.0790
MF	GO:0004970	Ionotropic glutamate receptor activity	1/11	19/18410	0.0112	0.0707
MF	GO:0042834	Peptidoglycan binding	2/11	18/18410	4.94E-05	0.0058

Abbreviations: BP, biological process; Bg ratio, background ratio; CC, cellular component; MF, molecular function.

**TABLE 3:** Gene set enrichment analysis (GSEA) in sciatica.

Description	NES	pvalue	p.Adjust	q value
REACTOME_NEUTROPHIL_DEGRANULATION	2.638027419	1E-10	2.78E-08	2.31E-08
KEGG_COMPLEMENT_AND_COAGULATION_CASCADES	1.91456794	0.00028	0.0083	0.0069
REACTOME_INTERFERON_GAMMA_SIGNALING	2.094977977	9.02E-07	6.10E-05	5.08E-05
KEGG_TOLL_LIKE_RECEPTOR_SIGNALING_PATHWAY	1.769730348	0.0007	0.0163	0.0136
KEGG_CYTOKINE_CYTOKINE_RECEPTOR_INTERACTION	1.560775876	0.0002	0.0085	0.0071
REACTOME_DNA_REPAIR	−1.628582411	3.71E-05	0.0016	0.0013
KEGG_CELL_CYCLE	−1.730620279	0.0004	0.0111	0.0092
KEGG_RIBOSOME	−2.509866873	1E-10	2.78E-08	2.31E-08
REACTOME_TRANSLATION	−2.633223202	1E-10	2.78E-08	2.31E-08

Abbreviations: NES, normalized enrichment score.

### 3.3 Construction of sciatica risk model

**FIGURE 6:**
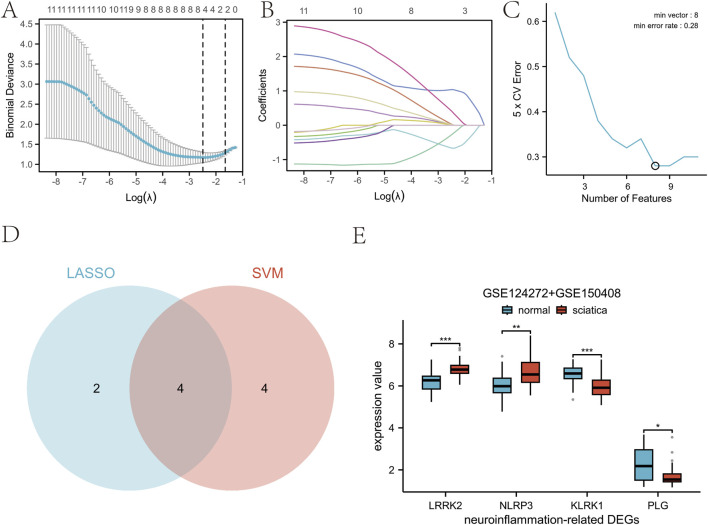
Identification and analysis of sciatica biomarkers based on machine learning. **(A)** Plot of binomial distribution bias versus log(λ) of LASSO regression. **(B)** Plot of coefficient paths of LASSO regression at different λ values. **(C)** Plot of the influence of the number of variables in the SVM-RFE method on the prediction performance of the model. **(D)** Venn plot of the selected variables of SVM-RFE versus LASSO model. **(E)** Box plot of different gene expression between normal and sciatica groups.

We analyzed the DEGs in the dataset using two algorithms. The LASSO regression identified six key genes (Figures 6A,B) (Supplementary Tables S6, S7), with the optimal regularization parameter (λ = 0.082924) selected through 10-fold cross-validation minimizing binomial deviance (Supplementary Table S8). The SVM machine learning algorithm identified eight key genes (Figure 6C; Supplementary Table S9), employing a radial basis function kernel with hyperparameters (cost = 0.5, gamma = 0.015625) optimized via 5-fold cross-validation, yielding a minimum cross-validation error rate of 0.28. A Venn diagram showed the key genes identified by both methods: *LRRK2*, *NLRP3*, *KLRK1*, and *PLG* (Figure 6D). Box plots showed notable disparities in the expression levels of four pivotal neuroinflammation-related genes between the normal and sciatica groups (Figure 6E).

**FIGURE 7:**
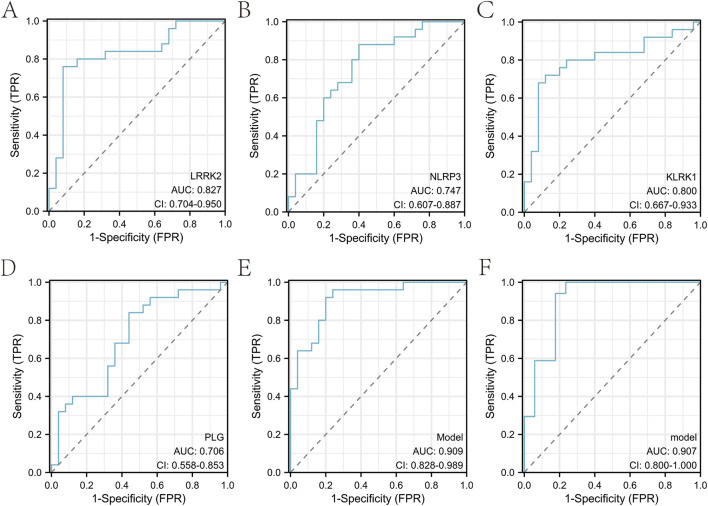
Evaluation of machine learning-assisted biomarkers in the diagnosis of sciatica. **(A–D)** Each panel presents the ROC curves of individual biomarkers for sciatica. **(E)** ROC curve of the four-gene model in the development phase. **(F)** ROC curve of the final model on the hold-out validation cohort.

The receiver operating characteristic (ROC) curve showed that a neuroinflammatio-n gene distinguished the normal from the sciatica group (Supplementary Table S10). The area under the curve (AUC) for each individual gene was derived from univariate ROC analysis using its normalized expression value as a continuous predictor. The area under the curve (AUC) and its 95% confidence interval for each gene were as follows: *LRRK2*: 0.827 (95% CI: 0.704-0.950) (Figure 7A); *NLRP3*: 0.747 (95% CI: 0.607-0.887) (Figure 7B); *KLRK1*: 0.800 (95% CI: 0.667-0.933) (Figure 7C); *PLG*: 0.706 (95% CI: 0.558-0.853) (Figure 7D). Following our two-stage analytical workflow, data from GSE124272 and GSE150408 were integrated for model development. Consensus hub genes (LRRK2, NLRP3, KLRK1, PLG) were identified from this combined dataset through the aforementioned LASSO and SVM-RFE analyses with cross-validation. A final diagnostic model was then constructed using only these four hub genes. The ROC curve of the final model was analyzed together with the aforementioned multiple biomarkers (Supplementary Table S11). This model achieved an AUC of 0.909 (CI = 0.828-0.989) in the development phase (Figure 7E). For the final, unbiased performance assessment, this diagnostic model, constructed using only the identified hub genes, was applied to the pre-specified, hold-out GSE150408 validation cohort. The model achieved an AUC of 0.907 (95% CI = 0.800-1.000) on this internal validation set (Figure 7F). A comprehensive set of performance metrics for this final diagnostic model, including accuracy, sensitivity, specificity, precision, F1-score, and the Matthews Correlation Coefficient (MCC) calculated at the optimal cut-off value, is provided in Supplementary Table S12.

**FIGURE 8:**
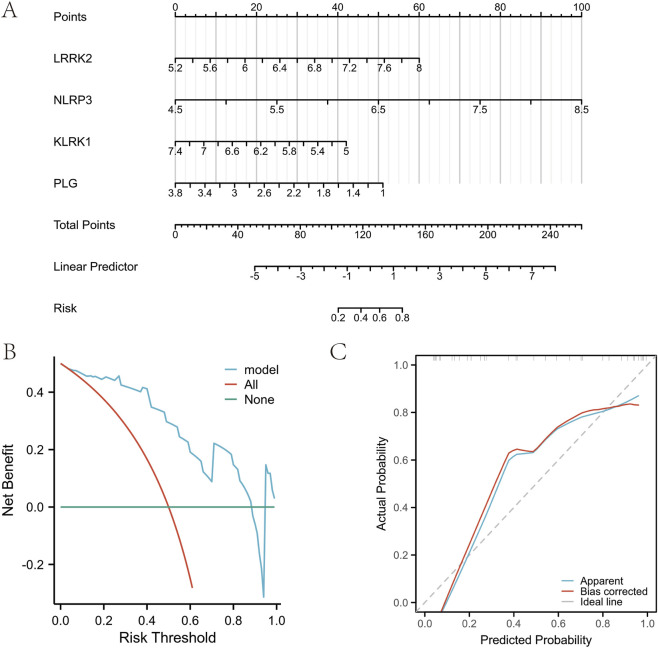
Construction of the sciatica risk model. **(A)** Standardized nomogram of biomarkers for predicting sciatica. **(B)** Decision curve analysis. **(C)** Calibration curve.

**FIGURE 9:**
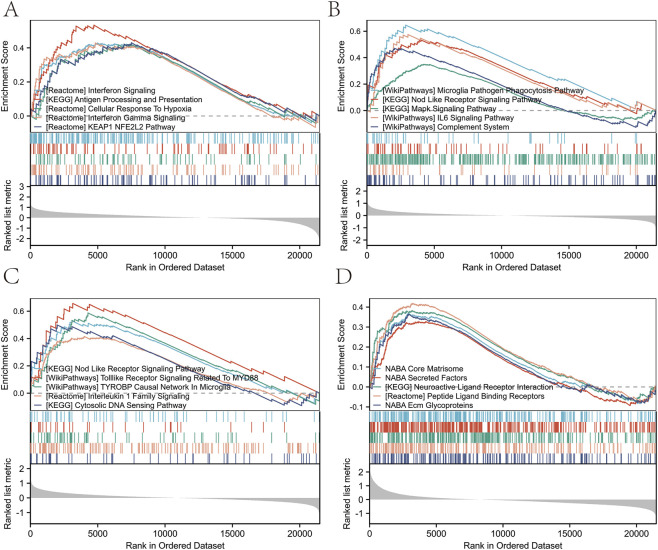
GSEA pathway analysis of high and low biomarker expression groups for machine learning screening. **(A)** GSEA pathway enrichment analysis associated with LRRK2 marker. **(B)** GSEA pathway enrichment analysis associated with NLRP3 marker. **(C)** GSEA pathway enrichment analysis of KLRK1 marker. **(D)** GSEA pathway enrichment analysis associated with PLG marker.

The contributions of the four key neuroinflammation genes to disease risk were evaluated using nomograms (Figure 8A). We also evaluated the clinical utility and prediction accuracy of the models using decision curve analysis and calibration curve analysis. The findings from the decision curve analysis revealed that employing the key genes for risk assessment offers considerable clinical advantages (Figure 8B). Furthermore, the calibration curve demonstrated a strong agreement between the predicted probabilities and the observed outcomes, indicating good model calibration when tested on the validation dataset (Figure 8C; Supplementary Table S13). Using GSEA, associations were identified between groups with high or low expression of four key genes and their corresponding pathological pathways. Among the key genes, *LRRK2* is associated with the WP microglia pathogen phagocytosis pathway (NES = 2.201, FDR <0.001) and the KEGG nod-like receptor signaling pathway (NES = 1.969, FDR <0.001) (Figure 9A). *NLRP3* is linked to the KEGG nod-like receptor signaling pathway (NES = 1.890, FDR <0.001) as well as the WP toll-like receptor signaling pathway related to MYD88 (NES = 2.047, FDR <0.001) (Figure 9B). *KLRK1* shows associations with the REACTOME cellular response to hypoxia (NES = 1.592, FDR <0.001) and REACTOME interferon signaling (NES = 1.867, FDR <0.001) (Figure 9C). Finally, *PLG* is connected to the NABA core (NES = 1.609, FDR <0.001) and the KEGG neuroactive ligand-receptor interaction pathway (NES = 1.666, FDR <0.001) (Figure 9D).

### 3.4 Immunological profile of sciatica

**FIGURE 10:**
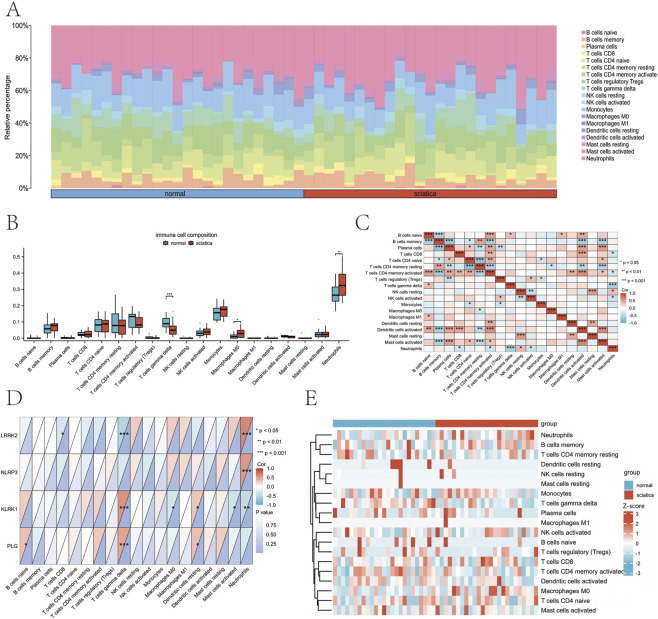
Features of immune cell infiltration in sciatica. **(A)** The estimated proportion of immune cells is shown as a bar chart. **(B)** Box plot of group comparison of immune cell composition. **(C)** Heat map of cell type correlation matrix. **(D)** Heat map of the correlation between biomarkers and immune cell types. **(E)** Clustering heatmap of immune cell types and biomarker expression levels.

This study examines changes in the immune cell composition of patients with sciatica using gene expression analysis specific to individual cell types (Figure 10A). We analyzed the immune cell composition between the control and sciatica groups. This analysis revealed numerous statistically meaningful distinctions, particularly in T cells gamma-delta, macrophages M0, and neutrophils. The proportion of M0 macrophages and neutrophils in the sciatica group was significantly greater than in the normal group (*p* < 0.05). Additionally, the proportion of gamma-delta T cells in the sciatica group decreased significantly compared to the normal group (*p* < 0.001) (Figure 10B). Spearman correlation analysis revealed a strong positive correlation between activated CD4 memory T cells and various cell types, including activated mast cells, dendritic cells (both activated and resting), naive CD4 T cells, CD8 T cells, plasma cells, and naive B cells (Figure 10C). *LRRK2*, *NLRP3*, *KLRK1*, and *PLG* correlate significantly with the prevalence of various immune cell types. *LRRK2* exhibited a positive correlation with neutrophils and a negative correlation with γδ T cells, CD8 T cells. NLRP3 showed a positive correlation with neutrophils. *KLRK1* demonstrated a positive correlation with γδ T cells, resting dendritic cells and macrophages, activated mast cells, and neutrophils. Similarly, *PLG* was positively correlated with γδ T cells and resting dendritic cells (Figure 10D). The heatmap shows the distribution of immune cells in the normal and sciatica groups (Figure 10E). To address potential limitations in neurological disease applications, we validated CIBERSORTx estimates using MCP-counter analysis. Both methods consistently identified elevated neutrophil infiltration in patients with sciatica, confirming the robustness of our findings across deconvolution platforms (Supplementary Figure S2).

### 3.5 Machine learning-driven identification of biomarkers and miRNA, TF, and small molecule drug action networks

**FIGURE 11:**
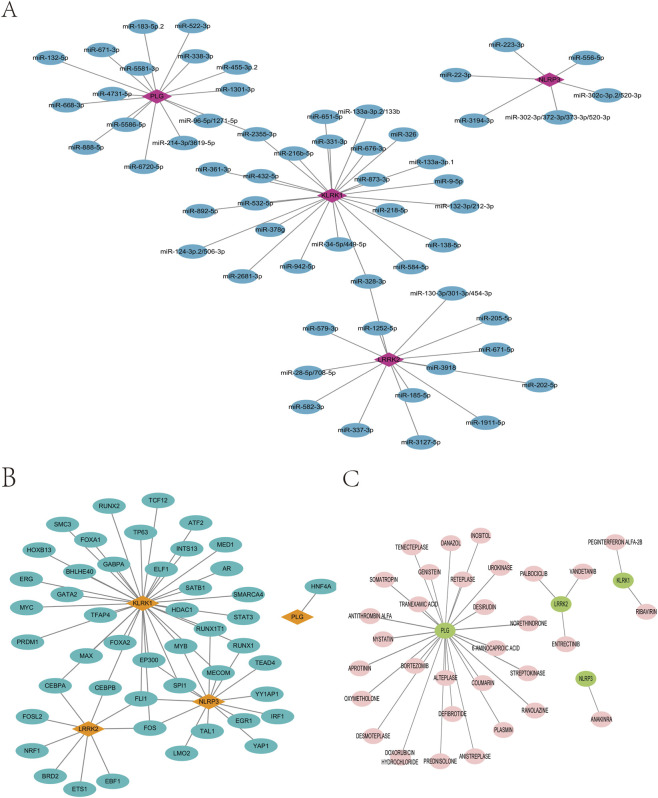
Machine learning-assisted screening of biomarkers with miRNA, TF, and small molecule drug interaction network. **(A)** biomarker and miRNA interaction network. **(B)** biomarker-transcription factor (TF) interaction network. **(C)** biomarker and small molecule drug action network.

A network of neuroinflammation-related genes involving mRNAs and miRNAs was created, consisting of four mRNAs and sixty miRNAs, with NLRP3 targeted by pro-inflammatory miR-22-3p and miR-223-3p, and KLRK1 showing the most extensive regulatory connectivity, suggesting complex post-transcriptional modulation of the neuroinflammatory signature (Figure 11A). The mRNA-TF network of neuroinflammation-related hub genes, comprising four mRNAs and fifty-nine TFs, was constructed using the ChIPBase v3.0 database, revealing distinct regulatory clusters wherein STAT3 and GATA2 targeted KLRK1, SPI1 and IRF1 regulated NLRP3, and HNF4A exclusively bound PLG, implicating coordinated activation of myeloid inflammation and interferon signaling pathways (Figure 11B). The DGIdb version 3.0.2 (https://www.dgidb.org) predicted potential drugs or small molecule compounds associated with four mRNAs and thirty-two small molecule drugs, notably identifying the FDA-approved IL-1 receptor antagonist Anakinra targeting NLRP3, multi-kinase inhibitors (Vandetanib, Entrectinib) targeting LRRK2, and fibrinolytic agents (Alteplase, Streptokinase) converging on PLG, thereby highlighting actionable therapeutic targets for sciatica intervention (Figure 11C).

### 3.6 Identification of four diagnostic gene expressions

**FIGURE 12:**
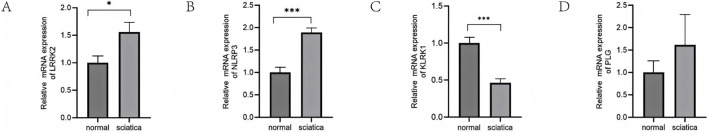
qRT-PCR results of neuroinflammatory biomarkers in patients with sciatica. **(A)** relative mRNA expression of LRRK2. **(B)** relative mRNA expression of NLRP3. **(C)** relative mRNA expression of KLRK1. **(D)** relative mRNA expression of PLG. (*p < 0.05, **p < 0.01, ***p < 0.001).

In addition, we measured the expression levels of *LRRK2*, *NLRP3*, *KLRK1*, and *PLG* in patients with sciatica and normal individuals using qRT-PCR. The study found that *LRRK2* (*p* < 0.05, Figure 12A) and *NLRP3* were significantly upregulated in sciatica samples compared to standard samples (*p* < 0.001, Figure 12B), while *KLRK1* was significantly downregulated (*p* < 0.001, Figure 12C). The expression of *PLG* tends to increase, but not significantly (*p* > 0.05, Figure 12D).

## 4 Discussion

Sciatica is a common form of peripheral nerve pain, but its causes are not fully understood; however, neuroinflammation is believed to play a key role (Zhang et al., 2023). Currently, diagnosis relies primarily on clinical symptoms, physical examinations, and imaging studies. However, these methods have limitations for tracking disease progression and predicting prognosis, underscoring the urgent need for new biomarkers to improve prognostic assessments and treatment guidance (Jungen et al., 2019). In recent years, the role of neuroinflammation in peripheral nerve pain has gained significant attention. Neuroinflammation is a core pathophysiological mechanism characterized by the overexpression of inflammatory factors and the activation of neuroimmune responses. These factors not only directly damage nerve fibers but also activate glial cells, further exacerbating the neuroinflammatory response, leading to the continuous transmission of pain signals and neurological dysfunction (Shi et al., 2024; Hu et al., 2020). Detailed research on neuroinflammation-related genes in sciatica is crucial for understanding its causes and for identifying new diagnostic biomarkers and treatment targets.

We used bioinformatics to identify DEGs from the GEO database datasets GSE124272 and GSE150408 to explore the relationship between neuroinflammation and sciatica. We identified significant changes in biological pathways associated with sciatica using GO functional enrichment and GSEA. Notably, although some gene overlap exists among functionally related pathways, applying stringent FDR correction (q-value <0.001) across all analyses ensured robustness against false positives, as detailed in Supplementary Table S14. The convergence of neuroinflamma-tory themes across independent databases (e.g., KEGG and Reactome) further supports the biological coherence of these findings. The results suggest that neuroinflammatory genes contribute cumulatively to multiple pathways related to sciatica.

In our study, we identified four differentially expressed neuroinflammatory genes- *LRRK2*, *NLRP3*, *KLRK1*, and *PLG*—through the innovative application of Lasso regression and SVM-RFE machine learning techniques. According to the ROC curve analysis, individual biomarkers showed good ability to distinguish between normal controls and sciatica patients. To further explore the upstream regulatory mechanisms and therapeutic potential of these four key genes, we performed an integrative network analysis encompassing miRNA-gene interactions, transcription factor (TF) regulation, and drug-gene associations. This computational approach yielded several mechanistically insightful and translationally relevant predictions. Notably, miR-22-3p and miR-223-3p were predicted to target NLRP3, while KLRK1 exhibited the most extensive miRNA connectivity, suggesting complex post-transcriptional modulation of the neuroinflammatory signature. Given the miR-22-3p′s established role as a negative regulator of inflammasome activity, this finding suggests a potential epigenetic mechanism in which reduced miRNA expression could disinhibit NLRP3-driven IL-1β production, thereby perpetuating sciatica pathogenesis. Furthermore, distinct regulatory clusters were identified wherein STAT3 and GATA2 targeted KLRK1, SPI1, and IRF1 regulated NLRP3 (implicating coordinated myeloid inflammation and interferon signaling), and HNF4A exclusively bound PLG. This convergence implicates specific transcriptional programs in the coordinated dysregulation of the neuroinflammatory gene set, providing testable hypotheses regarding disease mechanism. From a translational perspective, the drug-gene interaction network highlighted immediately actionable candidates, including the FDA-approved IL-1 receptor antagonist Anakinra, specifically targeting NLRP3; multi-kinase inhibitors (Vandetanib and Entrectinib), targeting LRRK2; and diverse fibrinolytic agents (such as Alteplase and Streptokinase) converging on PLG. This not only supports the biological plausibility of our identified targets but also proposes clinically translatable candidates for drug repurposing studies aimed at mitigating neuroinflammation in sciatica. In summary, these network analyses move beyond simple biomarker identification; they provide a mechanistic framework implicating specific miRNA-TF regulatory axes (e.g., miR-22-3p/NLRP3/SPI1-IRF1) and therapeutic avenues for future experimental validation, thereby significantly enhancing the mechanistic depth and translational value of our study. *LRRK2* is essential for nerve cell signaling, and abnormal levels of this protein can disrupt normal physiological functions and neuroinflammatory responses (Wang et al., 2021). It regulates inflammation-related pathways, such as mTOR, which affects nerve cells' sensitivity to inflammation (Nabar et al., 2022). *LRRK2* also influences the balance of the cytoskeleton, which affects nerve cell shape and function (Moehle et al., 2012). Additionally, it is a key gene linked to neurodegenerative diseases like Parkinson’s. Abnormal *LRRK2* activity triggers neuroinflammation by disrupting autophagy, leading to α-synuclein accumulation and the release of pro-inflammatory cytokines. It regulates mitochondrial function, increasing nerve cell sensitivity to inflammation and ROS production, which exacerbates neuroinflammation and cell damage (Liu et al., 2022). Furthermore, *LRRK2* affects synaptic plasticity and neurotransmission. Its abnormal activation leads to synaptic dysfunction, which disrupts neural signaling and contributes to a cycle of neuroinflammation and neurodegeneration (Mishra et al., 2025). The NLRP3 inflammasome is essential for neuroinflammation, as it activates interleukin-1β and interleukin-18, which increase nerve excitability and inflammation, thereby establishing a feedback loop. Activating the TRPV1 channel enhances NLRP3 assembly and inflammatory responses (Luo et al., 2011). In neuro-degenerative diseases such as Alzheimer’s, excessive *NLRP3* activation leads to pyroptosis in glial cells, exacerbating neuroinflammation and advancing disease progression (Mandal et al., 2025). Lower levels of *KLRK1* expression can weaken immune surveillance during neuroinflammation, impair NKG2D-mediated responses, and increase the susceptibility of neural cells to damage, which may worsen neuroinflammation and neural damage by altering the interactions between immune and neural cells (Walsh et al., 2008). *PLG* has a dual role in neuroinflammation, potentially worsening it by activating microglia and astrocytes (Reuland and Church, 2020), but may also protect against Alzheimer’s by degrading toxic proteins like Aβ (Oh et al., 2014). It influences immune responses and blood-brain barrier permeability (Shaw et al., 2017). In this study, qRT-PCR revealed elevated *PLG* expression in patients with sciatica patients compared with controls. While apparently contradicting bioinformatics findings, this discrepancy reflects clinically meaningful heterogeneity: 1) GSE124272 (lumbar disc prolapse) and GSE150408 (conservatively managed sciatica) represent distinct disease spectra; 2) Our surgical validation cohort (treatment-naïve discogenic sciatica) differed fundamentally from the conservatively managed GSE150408 cohort; 3) *PLG*’s role in extracellular matrix remodeling may be differentially activated in surgical versus managed care contexts. With technical controls rigorously validated, we interpret this not as a methodological artifact but as a biological insight suggesting that PLG expression may correlate with disease severity or treatment response—meriting explicit subgroup analysis in future studies.

Based on our findings, we employed standardized nomograms to evaluate the importance of each key gene in predicting disease risk. The analysis of decision curves indicated that integrating essential genes into risk assessment provides notable clinical advantages. Additionally, calibration curves showed good agreement between predicted probabilities and observed frequencies, indicating a well-calibrated model across independent datasets. This achievement represents a significant advance in predicting sciatica risk, underscoring our novel strategy for addressing complex biomedical problems through contemporary computational techniques. Thus, further exploration of these genes' functions could provide insights into the pathogenesis of sciatica.

We used the GSEA method to examine how different biomarker expression profiles relate to pathological pathways. The *KLRK1* gene showed significant enrichment in key pathways, including antigen processing and presentation, cellular response to hypoxia, interferon signaling, and oxidative stress response. This suggests that these processes may significantly contribute to the emergence and progression of sciatica. The analysis of the *LRRK2* gene revealed enrichment in pathways associated with microglial pathogen phagocytosis, NOD-like receptor signaling, MAPK signaling, IL-6 signaling, and the complement system. This reflects the possible influence of neuroinflammatory responses and immune regulation in the disease. The enrichment analysis of the *NLRP3* gene highlighted several important pathways, including the NOD-like receptor signaling pathway and the interleukin-1 family signaling pathway. These findings indicate the significance of inflammatory responses and glial cell activation in the disease. The analysis of the *PLG* gene revealed significant enrichment in pathways related to extracellular matrix core components, secreted factors, and neuroactive ligand-receptor interactions. These pathways may relate to key pathological features of neuroinflammatory diseases, including extracellular matrix remodeling and neuronal signaling.

Immune cell infiltration is crucial for the development and progression of sciatica (Starkweather et al., 2005). We used CIBERSORTx to analyze immune cell composition in patients with sciatica, enhancing our understanding of immune mechanisms and demonstrating advanced bioinformatics methods. The results revealed that sciatica patients had significantly higher counts of M0 macrophages and neutrophils compared to control subjects. In contrast, γδ T cell levels decreased. Li et al. reported similar findings in patients with lumbar disc herniation (Li et al., 2022). These studies highlight significant differences in immune cell numbers, suggesting changes in immune regulation in sciatica. An increase in M0 macrophages and neutrophils may indicate heightened inflammation, while a decrease in γδ T cells suggests an immune imbalance (Li et al., 2022; Zheng et al., 2022). Correlation analysis revealed strong positive associations between activated CD4 memory T cells and several immune cell types, including activated and resting mast cells, dendritic cells, naïve CD4 T cells, CD8 T cells, plasma cells, and naïve B cells. A significant positive correlation existed between activated dendritic cells and various immune cells, while neutrophils negatively correlated with γδT cells. *LRRK2*, *NLRP3*, *KLRK1,* and *PLG* correlated with immune cell abundance. *LRRK2* and *NLRP3* appear to drive neutrophilic inflammation, potentially through mediating neutrophil recruitment and activation. Conversely, *KLRK1* and *PLG* show protective associations with γδ T cells and dendritic cells, possibly contributing to immune regulation and tissue repair. The negative correlation between *LRRK2* and CD8^+^ T cells further suggests compromised adaptive immunity in the pathogenesis of sciatica. This suggests that *LRRK2*, *NLRP3*, *KLRK1*, and *PLG* may influence sciatica by regulating immune cells, indicating a need for further research on gene-immune cell interactions.

### 4.1 Limitation

Although this study presents important findings, it also has several notable limitations. First, the initial gene screening used a *p*-value threshold rather than a stricter FDR correction to retain an adequate number of candidate genes for downstream machine learning analysis. While this approach was necessary for our hypothesis-generating design and for the robustness of the final model, which was confirmed through rigorous machine learning regularization techniques, it may have increased the potential for false positives in the initial selection phase. Second, the study did not provide a thorough analysis of sciatica-related tissues, and without direct samples, our understanding of the genetic mechanisms underlying neuroinflammation is limited. Third, the machine learning validation employed an internal “leave-one-dataset-out” strategy: data from GSE124272 and GSE150408 were integrated for model development and feature selection via cross-validation, after which the entire, pre-specified GSE150408 cohort was held out exclusively for final performance estimation (AUC = 0.907). While this pre-defined, two-stage workflow rigorously separates model tuning from final evaluation to mitigate optimism bias, it remains an internal validation due to the use of the same data source for both purposes. The scarcity of additional public transcriptomic datasets for sciatica precluded fully independent external validation, which represents a limitation for the generalizability of the biomarker signature. Future studies should increase sample sizes and conduct multicenter research. Fourth, due to the heterogeneity of sciatica and the absence of clinical data, it was impossible to evaluate the relationship between risk indicators and patient stratification. Future research should incorporate detailed clinical data for a comprehensive analysis and interpretation. Fifth, the symptoms of patients and the expression of biomarkers vary, and future research needs to explore the characteristics of different subgroups to identify more specific biomarkers. Sixth, while the use of β-actin as a single reference gene in qRT-PCR experiments was substantiated by rigorous validation demonstrating stability across experimental conditions (including non-significant Cq value differences between groups and minimal technical variability), this approach may not represent the optimal strategy compared to employing multiple reference genes. We have addressed this methodological consideration in the discussion and will incorporate additional validated reference genes in future investigations to align with best practices. Seventh, regarding the assessment of batch effects, the Principal Component Analysis (PCA)-based metric (variance proportion on PC1+PC2) used in this study, while transparent and interpretable for our purpose, may not fully decompose variance components as robustly as advanced methods like Principal Variance Component Analysis (PVCA). Future studies employing multi-batch designs would benefit from implementing such integrated approaches for more granular analysis. Lastly, while this study primarily focused on gene expression analysis, future research should validate the functions of key genes using cell-based experiments and animal models. In summary, future research should increase sample sizes and combine histological and clinical data to more comprehensively elucidate the pathological mechanisms of sciatica.

## 5 Conclusion

In this study, we identified DEGs linked to neuroinflammation in patients with sciatica using the GEO database. We selected four key genes (*LRRK2*, *NLRP3*, *KLRK1*, *PLG*) for machine-learning-based diagnosis. These genes showed excellent predictive performance and could be promising diagnostic candidates.

## Data Availability

The original contributions presented in the study are included in the article/Supplementary Material, further inquiries can be directed to the corresponding author.
