# Supplementary figures and images for: Identification of gene expression signatures associated with neuroinflammation in discogenic sciatica using machine learning and experimental validation
Source: Front Genet. 2026 Mar 5;17:1666639. doi: 10.3389/fgene.2026.1666639 (PMC13000421; doi:10.3389/fgene.2026.1666639)

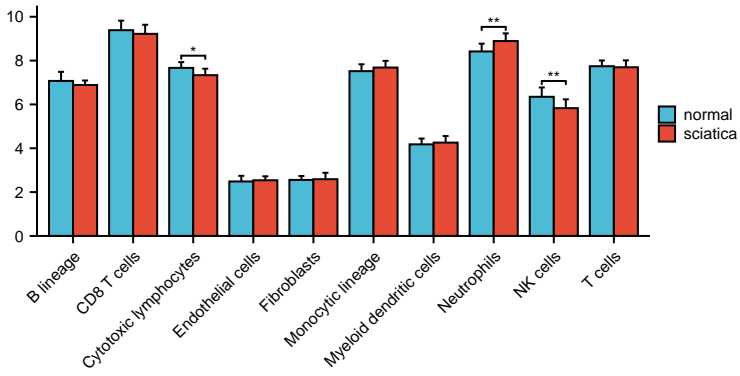

Supplement: Supplementary file 5 [file Image2.pdf]

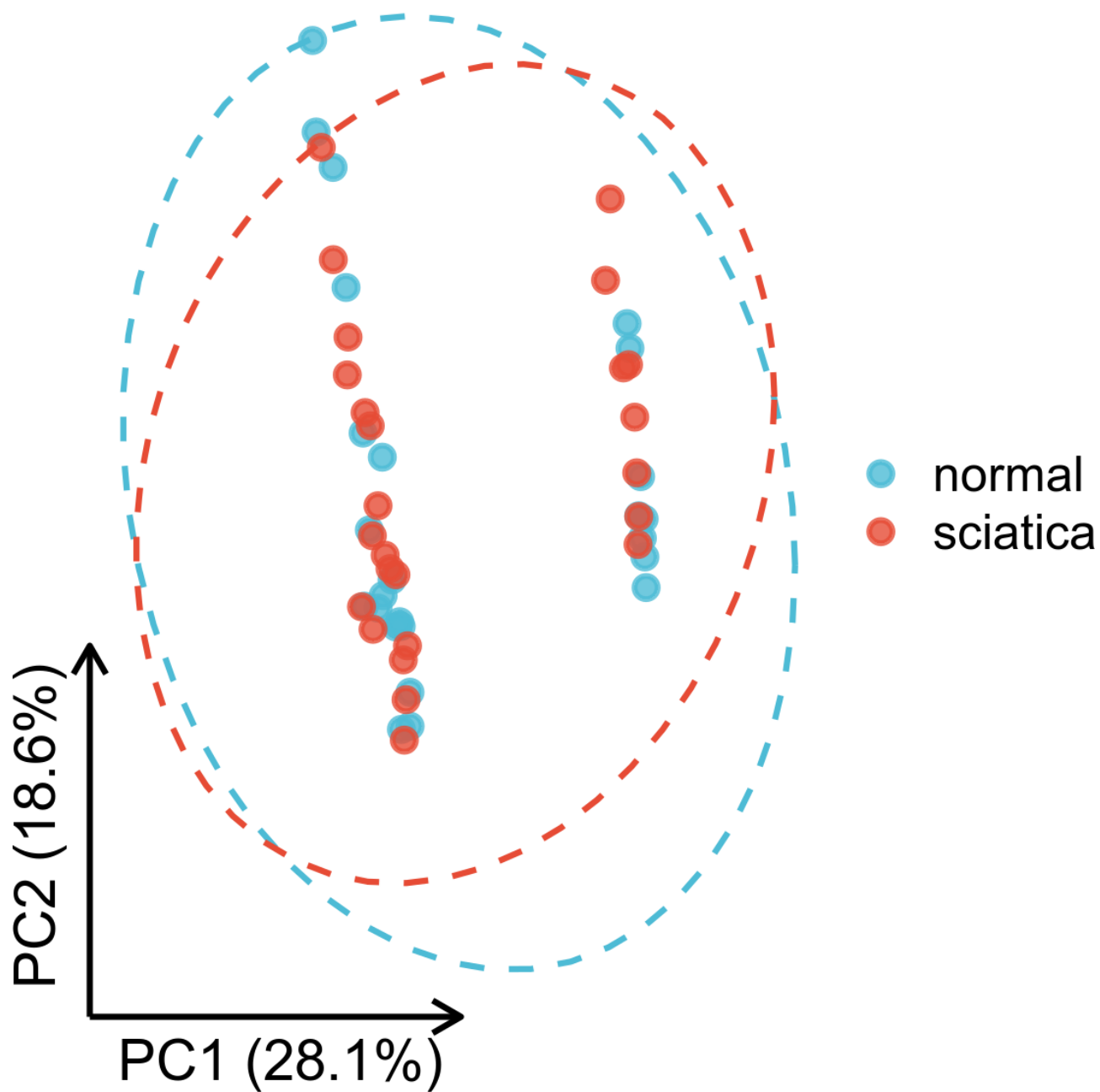

Supplement: Supplementary file 16 [file Image1.pdf]
